# Validity of caries data in the Swedish Quality Register (SKaPa) in a cohort of 19-year-olds in Region Värmland, Sweden

**DOI:** 10.2340/aos.v85.46895

**Published:** 2026-10-12

**Authors:** Tita Kirkinen, Gunilla Klingberg, Erik Hesselbom, Aron Naimi-Akbar

**Affiliations:** a Department of Pediatric Dentistry, Faculty of Odontology, Malmö University, Malmö, Sweden; b The Clinic of Pediatric Dentistry, Region Värmland, Sweden; c Health Technology Assessment – Odontology (HTA-O), Faculty of Odontology, Malmö University, Malmö, Sweden

**Keywords:** Dental caries, registries, validation study, adolescent

## Abstract

**Objective:**

The Swedish Quality Register for Caries and Periodontal Disease (SKaPa) is a national registry providing oral health data for children and adolescents. Since no validation has been performed on adolescents with permanent dentitions, the aim of this study was to evaluate SKaPa data for dental caries in 19-year-olds.

**Material and Methods:**

This diagnostic accuracy study included a random sample of 19-year-olds (*N* = 250) examined in 2014 in the Public Dental Service, Region Värmland. Clinical records served as the reference standard and SKaPa variables as the index test. Number of decayed and filled teeth (DFT), number of decayed, missing, and filled teeth (DMFT), missing teeth due to caries (MTC), missing teeth due to any reason (MTA), number of decayed teeth (DT), and number of filled teeth (FT) were evaluated. Agreement was evaluated using exact agreement, Cohen’s kappa, and intraclass correlation coefficients.

**Results:**

Data showed high agreement for DFT, DMFT, MTA, DT, and FT (kappa between 0.82 and 0.90; ICC 0.93–0.96). SKaPa underestimated caries by 6–7%. Among 155 participants with DFT/DMFT ≥1, only three had MTC, limiting evaluation.

**Conclusions:**

SKaPa caries data demonstrate high accuracy. However, as MTC could not be evaluated, DMFT should be used with caution in research at this point. Researchers should instead use DFT but be observant of the risk of an underdiagnosis of dental caries in SKaPa for 19-year-olds.

## Introduction

The use of registries in research and quality assessment is increasingly vital in dentistry, as such data enable descriptive analyses of health status, linkage studies to explore disease causation, and evaluations of treatment outcomes, including the impact of preventive measures targeting risk groups or entire populations. The Swedish Quality Register for Caries and Periodontal Disease (SKaPa) is currently the only quality registry containing data on oral health and dental care for children and adolescents in Sweden, underpinning both research and clinical quality assurance at the individual, regional, and national levels [1]. SKaPa is entirely based on the automated extraction of data from electronic dental records, including information from all dental visits, such as examinations, oral health status, and treatments, which are subsequently transferred to the registry. This differs from most other quality registries, where data entry is performed manually. All electronic dental record systems used in Sweden are compatible with SKaPa. The registry contains information on dental clinics, dental healthcare professionals, and patients. Patient-level data include personal identity number, sex, age, tooth-specific diagnoses and treatments, risk assessments for caries and periodontitis, and details of the dental care provided.

Registry-based research requires valid data – that is, an assurance that the registry information accurately reflects individuals’ true health status [2–6]. Although SKaPa is increasingly used in registry studies and informs both epidemiological surveillance and care evaluation at the dental-care-provider and regional levels, its validity has not been fully assessed. To date, two validation studies on dental caries in children aged 6 and 12 years, both conducted by the current authors, have been published [7, 8]. Both studies used data from Region Värmland’s Public Dental Service (PDS), which employs the Alfa eCare Clinics® (formerly Carita, Swedish Care®) electronic health record system. The studies showed good agreement regarding the number of decayed and filled teeth (dft/DFT) in primary/permanent dentition, respectively, but for extracted/missing teeth (e/M) due to caries in primary/permanent dentition, respectively, and for the number of decayed, extracted or missing, and filled teeth (deft/DMFT) in primary/permanent dentition, respectively, the agreement was poor, with lower agreement for 12-year-olds than 6-year-olds. Based on these results, the use of e/M and deft/DMFT as output data from the registry was discouraged. However, as the DMFT index is frequently used in research both in Sweden and in other countries, it is important to evaluate DMFT data from SKaPa in older age groups.

The causes for the poor agreement between the dental journal and SKaPa data for e/M and deft/DMFT are unclear but likely related to registration practices and human error. For example, in the mixed dentition, unerupted permanent teeth may be mistakenly recorded as missing due to extraction. This can happen, for example, when entering the reason for a tooth being missing into the dental record via the provided drop-down list. Other examples include orthodontic extractions, which can be incorrectly recorded as caries-related missing teeth if the recorder makes a mistake. This may seem trivial, but unfortunately this faulty information/status becomes irretrievable when it is registered in SKaPa, and as a consequence, the output data for the variable M (missing due to caries) from the registry are wrong. By extension, this also affects the DMFT index. The effect of such misregistrations when dental health is assessed as DMFT in a larger group cannot be assessed with certainty. It is unclear whether the problems with misregistrations persist at older ages. These findings emphasize the critical importance of accurate diagnosis and clinical documentation.

When using registry data in research or to report on oral health in a population, there is a risk of overestimating oral health status if the deft/DMFT index cannot be applied. When reports are limited to dft/DFT, the effect of missing one or several teeth due to caries will be lost with a risk of significantly misrepresenting the condition in individuals with severe caries. A clear example, albeit from the primary dentition, is that a 6-year-old who has lost all primary teeth due to caries would be recorded as having a dft score of zero, i.e. identical to that of a caries-free child. If the data for e/M were correctly captured in SKaPa and the deft index could be used, then the same child’s deft score would be 20 and thus show the child’s true disease burden and the real impact of the caries disease. Similar problems can occur for any patient regardless of age if the registrations of the underlying cause of e/M are incorrectly recorded. This issue raises concerns about the reliability of M and DMFT data for research and clinical follow-up, revealing a critical limitation to the registry’s validity.

Comprehensive validation across the full pediatric and adolescent age spectrum, including those with complete permanent dentition, is essential to enable accurate epidemiological research studies, evaluations of caries burden in various populations, and assessments of preventive and therapeutic interventions. Therefore, the aim of this study was to evaluate the validity of information on dental caries for 19-year-olds in the SKaPa registry. Specifically, it investigated the extent to which the registry data on DFT and DMFT correspond with the information found in clinical dental records. In addition, corresponding agreements were evaluated for carious teeth (DT), restorations (FT), and extractions (MTC (missing teeth due to caries) and MTA (missing teeth due to any reason). The age group was selected because it typically presents fully erupted permanent dentition, allowing for a comprehensive assessment of data quality that is relevant to both late adolescence and adult dental care.

This report was written in accordance with the Standards for Reporting of Diagnostic Accuracy (STARD) checklist [9].

## Methods

### Study design

This diagnostic accuracy study follows the PIRO framework:

- Population: The study focuses on 19-year-old adolescents residing in Region Värmland in 2014.
- Index test: Data from the Swedish Quality Register for Caries and Periodontal Disease (SKaPa): DFT, DMFT, decayed teeth (DT), filled teeth (FT), teeth missing due to caries (MTC), and teeth missing due to any reason (MTA) were included for the year 2014.
- Reference standard: Dental records of the same data listed above for the year 2014.
- Outcome measures: Agreement between SKaPa and dental records was assessed via Cohen’s kappa, the intraclass correlation coefficient (ICC), exact agreement, and differences in mean DFT and DMFT.

### Participants and setting

The study was carried out in Region Värmland, Sweden, a county with both rural and urban areas and a population of approximately 283,000 inhabitants. The PDS is the main provider of dental care for children and adolescents (approximately 92%) in the region. In 2014, 2580 19-year-olds were examined by PDS in Region Värmland. Official statistics show that 36% of the total Swedish population of 19-year-olds were caries-free (with a mean DFT of 2.34) in 2014. The corresponding figures for the same age group in Region Värmland were 39% caries-free and a mean DFT of 2.01 [10]. The study population consisted of 250 randomly selected 19-year-olds examined in 2014 within the PDS in Region Värmland, corresponding to approximately 10% of the Värmland population. This sample size mirrors those of previous validation studies conducted on 6- and 12-year-olds and was determined in consultation with statisticians to ensure sufficient variation in dental health, socioeconomic status, and demographics. For this study, 19-year-olds were chosen because epidemiological data have been routinely reported for this age group for over 25 years, and 19 years is the last age at which dental care is provided free of charge. The year 2014 was chosen to align with our earlier validation studies for younger age groups and because it corresponds to a time when all children in the region were still routinely examined yearly. Thus, the coverage of the dental records in relation to the underlying population was high. In later years, not all children have been offered annual dental examinations. Approximately 163,000 asylum seekers arrived in Sweden in 2015, of whom 70,384 (43%) were children aged 0–18 years. Of these children, 35,369 (50% of all children and 22% of all asylum seekers) were unaccompanied minors. In Värmland, 8884 asylum seekers were residing by the end of 2015, including 3880 children (43%); of these, 1006 (26% of all children and 11% of all asylum seekers) were unaccompanied minors. The substantial increase in the number of children requiring dental care placed considerable additional demands on dental services. Due to limited capacity, not all children could be offered dental examinations within the expected timeframe, and dental services had to prioritize acute dental needs and targeted screening of newly arrived children. These changes in the population receiving dental care, together with changes in the organization, capacity, and prioritization of dental services, may have affected the completeness and quality of dental documentation during this period. Consequently, from 2015 onwards, obtaining a representative sample for dental record validation studies became more challenging, as changes in dental service provision and examination coverage may have influenced which children were examined and represented in the dental records [11, 12].

### Test methods

An application for data extraction was submitted to the SKaPa registry. SKaPa identified 250 individuals (in a randomization procedure) who had a documented dental examination by PDS in 2014. In the next step, SKaPa provided unique case numbers to all identified patients. Output data comprising information about dental health (DFT, DMFT, MTA, MTC, DT, and FT) together with information on sex and the unique case number for each individual were sent to the researchers. SKaPa also sent personal identity numbers and allocated unique case numbers for all individuals to the PDS who retrieved the dental records for the year 2014 for all individuals. The PDS substituted all identity information from the dental records with the unique case number for each patient, after which copies of the full dental records, including clinical notes and dental status registrations up to December 31, 2014, were made available for the researchers. Thereafter, researchers extracted data on DFT, DMFT, MTA, MTC, DT, and FT for each individual from the dental records and linked this to the corresponding SKaPa data for the specific individual. Throughout the process, all data were anonymized for the researchers. In this study, dental caries was defined as a registration of manifest dental caries in the dental records made by the dentist responsible for the dental examination and was recorded at the tooth level. In a Swedish context, a carious lesion is registered as manifest if there is collapse of the tooth surface, loss of tooth substance, or if the lesion has passed the enamel–dentin boundary in dental radiographs.

Agreement was calculated using Cohen’s kappa and the ICC for DFT, DFMT, MTA, MTC, DT, and FT across the entire sample and specifically among those with at least one affected tooth.

### Analysis

The proportion of registrations showing exact agreement (i.e. identical measurements in both data sources) between the index (i.e. SKaPa) and the reference (i.e. the electronic dental records) was calculated and expressed as a percentage. To provide a more comprehensive understanding of the data, two additional measures of agreement between the index and reference were also included: Cohen’s kappa, reported along with the test statistic and standard error, and the ICC, presented with the test statistic and a 95% confidence interval. Mean differences between the electronic dental records and SKaPa for DFT and DMFT were also analyzed using Student’s *t*-test. All analyses were conducted using Stata 15 SE.

## Results

### Participants

The study included 250 19-year-olds (123 men, 127 women). One of these, a male with asylum-seeking status, was excluded due to incomplete data. Thus, the final population was 249 (122 men, 127 women).

### Test results

A total of 94 individuals (38%) were caries-free (DFT = 0), and the study group had a mean value for DFT of 2.08. The agreements between the electronic dental records and the SKaPa registry for DFT, DMFT, MTA, DT, and FT were very good, as assessed by Cohen’s kappa, ICC, and exact agreement. When data were analyzed for those with caries or fillings, the agreements were lower but still good (Tables 1 and 2).

**Table 1:** Agreement between data on dental caries from 19-year-old patients’ electronic dental records and the SKaPa registry, shown using Cohen’s kappa, standard error (SE), and % agreement.

Variable	*N* total	Cohen’s kappa	SE	Exact agreement (%)	*N* with score > 1	Cohen’s kappa	SE	Exact agreement (%)
DFT	249	0.86	0.03	0.89	155	0.82	0.034	0.85
DMFT	249	0.86	0.03	0.89	155	0.82	0.034	0.85
MTA	249	0.90	0.04	0.96	55	0.80	0.078	0.85
MTC	249	1	0.04	1	3	1	0.408	1
DT	249	0.87	0.05	0.96	45	0.73	0.096	0.84
FT	249	0.82	0.03	0.87	142	0.74	0.038	0.79

Notes: For DFT ≥ 1, DMFT ≥ 1, MTA ≥ 1, MTC ≥ 1, DT ≥ 1, and FT ≥ 1, the analyses only include cases where the value for DFT/DMFT/MTA/MTC/DT/FT exceeds 0 in the dental record.

DFT: number of decayed and filled teeth; DMFT: number of decayed, missing, and filled teeth; MTA: number of teeth missing for any reason; MTC: number of teeth missing due to caries; DT: number of decayed teeth; FT: number of filled teeth.

**Table 2:** Intraclass correlation coefficient (ICC) and 95% confidence interval (CI) of dental caries in 19-year-olds recorded in electronic patient dental records and the SKaPa registry.

Variable	*N* total	ICC	95% CI		*N* with score ≥ 1	ICC	95% CI	
DFT	249	0.95	0.94	0.96	155	0.93	0.90	0.95
DMFT	249	0.95	0.94	0.96	155	0.94	0.91	0.95
MTA	249	0.96	0.94	0.96	55	0.90	0.83	0.95
MTC	249	1	–	–	3	1	–	–
DT	249	0.96	0.95	0.97	45	0.92	0.86	0.96
FT	249	0.93	0.91	0.95	142	0.99	0.98	0.99

Notes: For DFT **≥** 1, DMFT **≥** 1, MTA **≥** 1, MTC **≥** 1, DT **≥** 1, and FT **≥** 1, the analyses only include cases where the value for DFT/DMFT/MTA/MTC/DT/FT exceeds 0 in the dental record.

DFT: number of decayed and filled teeth; DMFT: number of decayed, missing, and filled teeth; MTA: number of teeth missing for any reason; MTC: number of teeth missing due to caries; DT: number of decayed teeth; FT: number of filled teeth.

The agreement between the electronic dental records and the SKaPa registry for DFT scores showed a Cohen’s kappa of 0.86 (Table 1) and an ICC of 0.95 (Table 2). For DMFT, Cohen’s kappa was 0.86 (Table 1), and the corresponding ICC value was 0.95 (Table 2). The data for 221 of the 249 participants were entirely accurate regarding DFT and DMFT (exact agreement, 89%) (Table 1).

The mean difference between the electronic dental records and SKaPa was 0.12 for both DFT and DMFT and 0.22 when DFT/DMFT values of zero were excluded (Table 3).

**Table 3:** Information on DFT, DMFT, MTA, MTC, DT, and FT recorded from dental records (reference standard) of 19-year-old patients vs. SKaPa, with mean/min/max difference and *p*-value.

Variable	*N*	Dental record				SKaPa				Difference			
		Mean	SD	Min	Max	Mean	SD	Min	Max	Mean	Min	Max	*p*-value
DFT	249	2.08	2.79	0	21	1.96	2.68	0	21	0.12	–1	9	0.033
DFT ≥ 1	155	3.34	2.88	1	21	3.12	2.82	0	21	0.22	–1	9	0.011
DMFT	249	2.11	2.91	0	22	1.99	2.80	0	22	0.12	–1	9	0.033
DMFT ≥ 1	155	3.39	3.04	1	22	3.17	2.99	0	22	0.22	–1	9	0.011
MTA	249	0.52	1.16	0	6	0.49	1.12	0	6	0.04	–3	2	0.095
MTA ≥ 1	55	2.36	1.31	1	6	2.15	1.42	0	6	0.22	0	2	0.006
MTC	249	0.03	0.35	0	5	0.03	0.35	0	5	0	0	0	–
MTC ≥ 1	3	2.67	2.08	1	5	2.67	2.08	1	5	0	0	0	–
DT	249	0.30	0.82	0	7	0.30	0.81	0	7	0.004	–1	2	0.782
DT ≥ 1	45	1.67	1.21	1	7	1.58	1.27	0	7	0.09	–1	2	0.209
FT	249	1.76	2.40	0	16	1.57	2.19	0	16	0.18	–1	8	< 0.001
FT ≥ 1	142	3.08	2.46	1	16	2.74	2.29	0	16	0.35	–1	8	< 0.001

Notes: For DFT ≥ 1, DMFT ≥ 1, MTA ≥ 1, MTC ≥ 1, DT ≥ 1, and FT ≥ 1, the analyses only include cases where the value for DFT/DMFT/MTA/MTC/DT/FT exceeds 0 in the dental record.

DFT: number of decayed and filled teeth; DMFT: number of decayed, missing, and filled teeth; MTA: number of teeth missing for any reason; MTC: number of teeth missing due to caries; DT: number of decayed teeth; FT: number of filled teeth.

A total of 155 individuals had a DFT/DMFT score greater than zero. Of these, only three had teeth missing due to caries (MTC ≥1). To assess the impact of extracted or missing teeth, as well as dental caries, on the accuracy of the SKaPa data, Cohen’s kappa, the exact agreement, and the ICC were calculated for individuals with caries (where DFT, DMFT, MTA, MTC, DT, and FT values exceeded 0 in the dental records), as shown in Tables 1–3.

## Discussion

The results of this study show an almost perfect agreement between the electronic dental records and the SKaPa registry for DFT and DMFT scores as well as for DT, FT, and MTA. However, an analysis of the differences in mean DFT and DMFT revealed some underestimation of dental caries by SKaPa. As the study population had good oral health, with very few teeth extracted due to caries, the variable of MTC could not be adequately validated, which affects the interpretation of DMFT. Although the statistical agreements were good for DMFT, the uncertainty of MTC implies that DMFT should be used with caution. For that reason, it is better to use DFT in research. More studies are still needed to fully evaluate MTC and DMFT.

In this study, the 19-year-old age group was chosen because epidemiological data have been routinely reported for 19-year-olds on both the regional and national levels for more than 25 years. The age also represents the last age at which dental care is provided free of charge in Sweden (with an exception for the period 2017–2025 when young adult up to 23 had free care). The year 2014 was selected to align with our earlier validation studies in younger age groups and corresponded to a period when children in the region were still routinely examined annually. This cohort also provided the highest examination coverage, thereby ensuring a representative study population. As the objective of this study was to validate the agreement between data recorded in dental records and the corresponding data transferred to SKaPa, rather than to estimate caries prevalence, the choice of study year is not expected to substantially affect the validity assessment. The recording of caries in the electronic dental records and the transfer of these data to SKaPa followed the same general principles as those used today, supporting the continued relevance of the findings for current use of the register. Further, in 2014, dental examinations reached 81% of all 19-year-olds in Sweden, while this dropped to around 60% in recent years, and in the year 2024, only 39% of the age group was examined by the PDS in Region Värmland [13]. This study used a random sample of all 19-year-olds who had a dental examination in 2014. This was based on the assumption that future studies using data from SKaPa would likely retrieve data for precisely random samples. When comparing dental health data for Region Värmland with national data [10], we saw that the patients in Värmland were on par with national data. Furthermore, results showed that the dental health in the present study sample was similar in terms of both percentage caries-free and mean DFT to the background population. In fact, the study sample even displayed somewhat more caries than the total group of 19-year-olds in Värmland. From a dental health perspective, it is fair to conclude that the study population mirrors the age group at both national and Region Värmland levels. These are all strengths of the present study.

The results of the present study, together with those of two previous studies [7, 8], are promising as they clearly show that the SKaPa registry can be used for research on dental caries in children and adolescents. This is an important finding, since research using registries is a growing field in which the quality of the studies is entirely dependent on access to verified, high-quality data from registries [2–5, 14]. There is still a lack of studies examining the accuracy of SKaPa’s data for dental caries in adults, although a recent study on periodontal disease data from SKaPa in adults reported good validity [15].

In the present study, the data from SKaPa differed from the dental records in that the latter reported higher numbers of caries, suggesting that SKaPa tends to report a somewhat better dental health picture. In other words, at the group level, individuals appear to be healthier in the SKaPa data (approximately 6–7% lower DFT and DMFT). This difference should be kept in mind both when conducting research and when using output data from SKaPa for dental health assessments and to plan dental care at a regional and national level. The population may have more caries than displayed in SKaPa data; in addition, caries disease is usually unevenly distributed within the population. The impact of the unequal distribution of dental caries on the population mean value was not studied in the present report. However, the kappa and ICC values decreased when healthy individuals were excluded.

The issue of underdiagnosis in SKaPa data needs to be problematized, as there is a risk of underestimating the prevalence of caries disease in the population. To address this issue, we suggest dividing reports on caries epidemiology into one for the total population and another for those affected by the disease. Moreover, we recommend calculating and reporting mean values only among individuals with caries and excluding caries-free individuals to obtain a more accurate picture of the affected population. This approach could provide a better basis for planning oral health interventions and for presenting dental health data in population-based reports.

For MTC, complete agreement was observed between SKaPa and the dental records; however, this was based on only three individuals, making it impossible to draw firm conclusions regarding the validity of this variable. The limited number of MTC cases is attributable to the low prevalence of extractions due to caries, reflecting the generally good oral health of the population. This resulted in a floor effect, with limited variability and insufficient numbers to robustly assess the accuracy of the MTC component. Further validation in a substantially larger population is therefore warranted. As it was not possible to fully evaluate MTC in the present study, and the fact that MTC constitutes an important component of DMFT, it is difficult to argue that DMFT is a fully valid output variable from SKaPa at this point. Despite the high agreement in terms of kappa and ICC values regarding DMFT, the underlying issues with MTC remain. Based on this, we recommend that researchers use DFT, DT, and FT for registry-based studies. If they wish to study tooth extractions, it is possible to retrieve output data from SKaPa for this based on the specific treatment codes for extractions that are used in the Swedish dental care system. This alternative has proved to be successful when analyzing dental health and dental care for children in out-of-home care in Sweden [16].

The reason for the differences between SKaPa and the dental records is not known. However, a transition to a new electronic dental record system was implemented in Region Värmland in 2010. During this process, previous dental restorations were not consistently recorded in the dental status section but were instead documented merely as prior dental treatments. Consequently, some historical treatments were not captured in the system. While this may be one reason for the recorded differences, it should be noted that there is seldom 100% agreement in validation studies. From that perspective, the kappa and ICC values found in this study can be considered high.

Several other regions are currently undergoing changes in the procurement of electronic dental record systems, and the validity of SKaPa data for dental caries has not been evaluated in other regions. As the transfer of data from dental records to SKaPa is automatized and these procedures are followed and continuously evaluated by the SKaPa administration, the risk for variation in validity is considered to be low. In our studies so far, we have analyzed data at the tooth level rather than the tooth surface level; moreover, our investigations have been limited to 6-, 12-, and 19-year-olds living in Värmland, which has a relatively healthy pediatric population. Consequently, the situation of other age groups and those in other regions remains unknown. Therefore, we believe that the SKaPa registry would benefit from being validated in populations with higher caries prevalence and in regions using other dental record systems. Furthermore, it is important to formulate a clear and specific research question when requesting data from SKaPa, involve the research specialists at SKaPa in the data collection, and discuss the data and purposes with them, as is recommended for other health registries. Doing so will ensure that the correct and age-appropriate variables are obtained from SKaPa, increasing the quality of the subsequent research.

To conclude, this study demonstrated very good agreement between the dental caries data in dental records and SKaPa for DFT and DMFT as well as for DT, FT, and MTA in 19-year-olds. Despite this, researchers are advised to avoid DMFT as an output variable from SKaPa based on the fact that MTC could not be fully evaluated in the present study. Instead, the use of DFT is encouraged. Combined with findings from previous studies, the results in this study indicate that SKaPa data on dental caries, foremost dft/DFT, in children and adolescents are sufficiently accurate for research purposes. Nonetheless, a potential risk of underdiagnosis of dental caries in SKaPa data among 19-year-olds should be taken into consideration.

## Data Availability

Researchers may apply to receive the datasets used and/or analyzed from the corresponding author upon reasonable request.

## References

[1] von Bultzingslowen I, Ostholm H, Gahnberg L, Ericson D, Wennstrom JL, Paulander J. Swedish quality registry for caries and periodontal diseases – a framework for quality development in dentistry. Int Dent J. 2019;69(5):361–8. 10.1111/idj.12481 PMC6790561 31001827

[2] Ludvigsson JF, Andersson E, Ekbom A, Feychting M, Kim JL, Reuterwall C, et al. External review and validation of the Swedish national inpatient register. BMC Public Health. 2011;11:450. 10.1186/1471-2458-11-450 PMC3142234 21658213

[3] Sorensen HT, Sabroe S, Olsen J. A framework for evaluation of secondary data sources for epidemiological research. Int J Epidemiol. 1996;25(2):435–42. 10.1093/ije/25.2.435 9119571

[4] Prang KH, Karanatsios B, Verbunt E, Wong HL, Yeung J, Kelaher M, et al. Clinical registries data quality attributes to support registry-based randomised controlled trials: a scoping review. Contemp Clin Trials. 2022;119:106843. 10.1016/j.cct.2022.106843 35792338

[5] Rosén M. National health data registers: a Nordic heritage to public health. Scand J Public Health. 2002;30(2):81–5. 10.1080/140349401753683444 12028856

[6] Ljung R, Lundgren F, Appelquist M, Cederlund A. The Swedish dental health register – validation study of remaining and intact teeth. BMC Oral Health. 2019;19(1):116. 10.1186/s12903-019-0804-7 PMC6580593 31208416

[7] Mensah T, Tranaeus S, Cederlund A, Naimi-Akbar A, Klingberg G. Swedish quality registry for caries and periodontal diseases (SKaPa): validation of data on dental caries in 6- and 12-year-old children. BMC Oral Health. 2021;21(1):373. 10.1186/s12903-021-01705-x PMC8305535 34301237

[8] Kirkinen T, Naimi-Akbar A, Cederlund A, Tranaeus S, Carlson C, Klingberg G. Accuracy of the Swedish quality registry for caries and periodontal diseases (SKaPa) – evaluation in 6- and 12-year-olds in the region of Varmland, Sweden. Acta Odontol Scand. 2023;81(8):615–21. 10.1080/00016357.2023.2235422 37470405

[9] Bossuyt PM, Reitsma JB, Bruns DE, Gatsonis CA, Glasziou PP, Irwig L, et al. STARD 2015: an updated list of essential items for reporting diagnostic accuracy studies. Br Med J. 2015;351:h5527. 10.1136/bmj.h5527 PMC4623764 26511519

[10] Socialstyrelsen. Karies hos barn och ungdomar. Epidemiologiska uppgifter för år 2014. (in Swedish). [National Board of Health and Welfare. Caries in children and adolescents. Epidemiological data for year 2014] [Internet]. [cited 2026 Aug 28]. Available from: www.socialstyrelsen.se/contentassets/62bb78e73423436ca7cc0217bef1c6da/2015-11-36.pdf

[11] Region Värmland. Flyktingmottagandet i Värmland. En ögonblicksbild från hösten 2015 [Internet]. 2017 [cited 2026 Aug 28]. Available from: https://www.regionvarmland.se/download/18.6b8233917a75a7b42c20c32/1626698679405/Rapport%2020%3A%20Flyktingmottagandet%20i%20V%C3%A4rmland%20-%20En%20%C3%B6gonblicksbild%20fr%C3%A5n%20h%C3%B6sten%202015.pdf

[12] Socialstyrelsen. Hälso- och sjukvård och tandvård till asylsökande och nyanlända [Healthcare and dental care for asylum seekers and newly arrived migrants]. Slutrapport oktober 2016 (in Swedish). [National Board of Health and Welfare; 2016] [Internet]. [cited 2026 Aug 28]. Available from: https://www.socialstyrelsen.se/contentassets/2fc3409761124ba78aa846cdd9c5e736/2016-10-13.pdf

[13] Socialstyrelsen. Karies bland barn och ungdomar – 2024. Art. nr: 2025-6-9683 (in Swedish). [National Board of Health and Welfare. Caries in children and adolescents]. 2024. [cited 2026 Aug 28]. Available from: http://www.socialstyrelsen.se/contentassets/62bb78e73423436ca7cc0217bef1c6da/2015-11-36.pdf

[14] Everhov AH, Frisell T, Osooli M, Brooke HL, Carlsen HK, Modig K, et al. Diagnostic accuracy in the Swedish national patient register: a review including diagnoses in the outpatient register. Eur J Epidemiol. 2025;40(3):359–69. 10.1007/s10654-025-01221-0 PMC12137447 40140143

[15] Persson P, Bladh M, Teleka S, Milosavljevic A, Gustafsson N, Jaghagen EL, et al. Using dental register information and questionnaire data to assess periodontitis in large cohort studies. J Clin Periodontol. 2025;52(11):1529–39. 10.1111/jcpe.70015 PMC12531359 40827525

[16] Kirkinen T, Naimi-Akbar A, Cederlund A, et al. The Swedish out-of-home care children cohort (SweOHC) – evaluation of dental health and dental care. BMC Oral Health. 2025;25:1320. 10.1186/s12903-025-06389-1 PMC12344838 40797193

